# Dr. Teal’s Invalid Bed Attachment

**Published:** 1869-02

**Authors:** 


INVALID BED ATTACHMENT.—We have never used the Attachment refered to, but from an examination of it we have no doubt of its utility. It claims to be simple, cheap, and easily adjusted to any ordinary bed. We shall say more when we shall have tried it.

